# Vitamin A and E Homologues Impacting the Fate of Acrylamide in Equimolar Asparagine-Glucose Model System

**DOI:** 10.3390/antiox10070993

**Published:** 2021-06-22

**Authors:** Su Lee Kuek, Azmil Haizam Ahmad Tarmizi, Raznim Arni Abd Razak, Selamat Jinap, Maimunah Sanny

**Affiliations:** 1 Department of Food Science, Faculty of Food Science and Technology, Universiti Putra Malaysia, UPM, Serdang 43400, Selangor, Malaysia; suleekuek23@gmail.com (S.L.K.); jinap@upm.edu.my (S.J.); 2 Product Development and Advisory Services Division, Malaysian Palm Oil Board, 6, Persiaran Institusi, Bandar Baru Bangi, Kajang 43000, Selangor, Malaysia; raznim@mpob.gov.my; 3 Laboratory of Food Safety and Food Integrity, Institute of Tropical Agriculture and Food Security, Universiti Putra Malaysia, UPM, Serdang 43400, Selangor, Malaysia

**Keywords:** acrylamide, Vitamin A, Vitamin E, asparagine-glucose model system, heating

## Abstract

This study aims to evaluate the influence of Vitamin A and E homologues toward acrylamide in equimolar asparagine-glucose model system. Vitamin A homologue as β-carotene (BC) and five Vitamin E homologues, i.e., α-tocopherol (AT), δ-tocopherol (DT), α-tocotrienol (ATT), γ-tocotrienol (GTT), and δ-tocotrienol (DTT), were tested at different concentrations (1 and 10 µmol) and subjected to heating at 160 °C for 20 min before acrylamide quantification. At lower concentrations (1 µmol; 431, 403, 411 ppm, respectively), AT, DT, and GTT significantly increase acrylamide. Except for DT, enhancing concentration to 10 µmol (5370, 4310, 4250, 3970, and 4110 ppm, respectively) caused significant acrylamide formation. From linear regression model, acrylamide concentration demonstrated significant depreciation over concentration increase in AT (Beta = −83.0, *R*^2^ = 0.652, *p* ≤ 0.05) and DT (Beta = −71.6, *R*^2^ = 0.930, *p* ≤ 0.05). This study indicates that different Vitamin A and E homologue concentrations could determine their functionality either as antioxidants or pro-oxidants.

## 1. Introduction

Acrylamide is considered a heat-induced contaminant generated from carbohydrate-based foods when subjected to thermal treatments. This unwanted compound was first discovered in foods in 2002 and is currently classified as a probable carcinogen (Group 2A) by the International Agency for Research on Cancer (IARC) [1]. Acrylamide can be developed through Maillard reaction in the presence of two precursors, namely free asparagine and reducing sugars when food is prepared at high temperature [2]. Carbonyl scavenging is proposed to effectively reduce or prevent acrylamide formation by interrupting the critical steps or scavenging key intermediates on the pathways to acrylamide formation [3]. Antioxidants may scavenge these free radicals and decrease the formation of acrylamide. Vitamin E is one of the natural fat-soluble vitamins present in vegetable oils and fats. This phytonutrient can be classified into two isomers, namely tocopherols and tocotrienols [4]. Tocopherols mainly consist of four homologues, i.e., α-tocopherol (AT), β-tocopherol, γ-tocopherol, and δ-tocopherol (DT). Tocotrienols can be also categorized into four homologues encompassing α-tocotrienol (ATT), β-tocotrienol, γ-tocotrienol (GTT), and δ-tocotrienol (DTT) [5]. Vitamin E plays an essential role in inhibiting oxidation by slowing down the rate of hydroperoxides formation (primary oxidation) and subsequently reduces the formation of secondary constituents such as aldehyde [6]. Vitamin A is another form of the fat-soluble vitamin in vegetable oils and fats. Such vitamin is often found in crude palm oil at a considerable level between 500 and 1000 ppm [7]. In the form of α- and β-carotenes (BC), Vitamin A has been shown to have the ability to remove singlet oxygen and reduces peroxyl radical [8]. BC exhibits twice the Vitamin A activity (expressed in retinol equivalent) of alpha-carotene [7]. Kushairi et al. [9] coined that tocotrienols and BC have shown to possess potential health-promoting effects on humans.

Most studies in the literature have focused on the eliminating effects of water-soluble antioxidants on acrylamide [3,10]. Although research on the effect of fat-soluble antioxidants on acrylamide formation is scarce, few studies reported using fat-soluble antioxidants to minimize acrylamide formation. Li et al. [11] showed that the addition of Vitamin E (0.1 mg kg^−1^) to a cookie formula reduced acrylamide formation by 49.6%. In another study, Kamarudin et al. [12] reported that the effect of the fat-soluble antioxidants in Carotino red palm oil was more apparent when a prolonged frying time was used for 10 consecutive frying sessions of French fries. Further, Kuek et al. [13] observed acrylamide concentration in French fries decreased up to the 48th frying cycle, using red palm super olein (industrial grade). It is believed that fat-soluble antioxidants (i.e., BC and Vitamin E) affect acrylamide formation, which can be attributed to the ability of fat-soluble antioxidants with different structures or functional groups to react with acrylamide precursors, with intermediates of the reaction or with acrylamide itself, leading to either reducing or promoting effects [14]. Acrylamide is generated through a complex process that forms free radicals and many intermediate products during the reaction; therefore, the presence of antioxidants helps to prevent the free radical formation or hinder the attack of acrylamide from free radicals through extinction resulting in inhibition of acrylamide formation [11]. However, the discordant effect of antioxidants on the occurrence of acrylamide has been reported in the literature. Although Vitamin E has shown inhibitory effects on acrylamide in a cookie model, it was found to promote the meat model and beef nuggets [11,15,16]. Another study shows that Vitamin E, in the form of AT, distorted the acrylamide formation in the fried snack model [17]. The authors concluded that Vitamin A in the form of retinol exhibited a slight inhibitory effect on acrylamide. Nevertheless, the effect of BC on acrylamide has yet to be elucidated in any context.

There are a few reported studies utilizing model systems with known substrates or intermediate products to understand the effect of antioxidants on acrylamide behavior [18,19]. The advantage of model systems is that relevant information is obtained about the actual mechanisms of the reaction, before proceeding to work on more realistic systems (i.e., food system) [20]. Antioxidants could limit the accumulation of carbonyls and thus inhibit acrylamide formation [18]. Inequality in the role of Vitamin E towards acrylamide behavior can be due to the different concentrations and homologues applied in the model system. As such, the reactivity of individual homologues, in their simplest form, at various concentrations requires further investigation to attest to acrylamide formation in the model system. Therefore, the objective of this study was to examine the effect of different concentrations of Vitamin A and E homologues on the formation of acrylamide in asparagine-glucose model system.

## 2. Materials and Methods

### 2.1. Chemicals and Reagents

Labeled [13C3]-acrylamide (99% isotopic purity, 1 mg/mL) was purchased from Cambridge Isotope Laboratories Inc. (Andover, MA, USA). Acrylamide (purity > 99%), D-(+)-glucose (purity ≥ 99.5%), L-asparagine (purity ≥ 98%), (+)-α-tocopherol (purity ≥ 96%), β-carotenes (purity ≥ 97%), and δ-tocopherol (purity ≥ 97%) were obtained from Sigma-Aldrich (St. Louis, MO, USA). The α-tocotrienol (purity 88%), δ-tocotrienol (purity 59%), and γ-tocotrienol (purity 95%) were obtained from ExcelVite Sdn. Bhd. (Chemor, Malaysia). Bromine (purity 99.99%), triethylamine, ethyl acetate, hexane, sodium sulphate anhydrous, silica gel 60 (0.063–0.200 mm, 70–230 mesh, ASTM), and sodium thiosulphate were acquired from Merck (Darmstadt, Germany). Silicone oil was purchased from Systerm (Shah Alam, Malaysia). All chemicals were of analytical grade. Ultra-pure water, i.e., 18.2 MΩ-cm, was utilized (Purelab Classic U.V., ELGA LabWater, High Wycombe, UK) throughout the research.

Acrylamide stock solution (1000 µg/mL) and 13C3-labeled acrylamide (4 µg/mL) were prepared following the dilution procedure. Two working acrylamide standards at 1 and 10 µg/mL were prepared from the stock solution (1000 µg/mL). These working standards were then used to prepare series of calibration standards at 0, 10, 50, 100, 250, and 500 ng/mL. Internal standard comprising 200 ng/mL of 13C3-labeled acrylamide was prepared from a stock solution of 4 µg/mL. One liter of bromination reagent was obtained by mixing 200 g of potassium bromide in a solution containing 160 mL of saturated bromine water, 10 mL of hydrobromic acid, and 0.5 L of ultra-pure water. All reagents were kept in a capped dark amber bottle at 4 °C.

### 2.2. Asparagine-Glucose Model System

Equimolar asparagine-glucose model system was established following the procedure from Ou et al. [19] with slight modification. The model system was first prepared from a mixture containing 10 µmol of each asparagine and glucose, and 1 µmol of BC was homogenized with 1 g of silica gel. The model system was then heated in an oil bath (Memmert, Germany) at 160 °C for 20 min before cooling at −20 °C for subsequent acrylamide analysis. A similar procedure was repeated for the remaining homologues (AT, DT, GT, ATT, DTT, and GTT). A similar procedure was also applicable for the increased concentration (10 µmol) for each homologue. The model system that contained only a mixture of asparagine and glucose with the absence of any vitamin homologues served as control.

### 2.3. Acrylamide Analysis

#### 2.3.1. Sample Extraction

Sample extraction was performed following the method established by Sanny et al. [21] with minor modification. About 0.5 g of sample was first weighed into a 50 mL capacity centrifugal tube containing a mixture of 10 mL of ultra-pure water and 200 ng/mL of 13C3-labeled acrylamide as internal standard. The mixture was then shaken at 256 pulses/min for 30 min using a vertical shaker (RS-1, Jeio Tech Co., Gyeonggi-do, Korea) and centrifugation using a refrigerated centrifugation system (3–18 K Sigma, Gillingham, Dorset, UK) at 10,960 RCF and 4 °C for 30 min before transferring 5 mL of supernatant into a capped glass bottle. The elute and standards of different concentrations (0, 10, 50, 100, 250, 500 µg/mL) were allowed to react with 15 mL bromination reagent for 2 h at 4 °C, followed by the addition of 0.7 M sodium thiosulphate until the yellowish color faded.

Approximately 4 g of anhydrous calcined sodium sulphate was further added and stirred until all powder dissolved. The mixture was transferred into another centrifuge tube for double extraction using ethyl acetate and hexane (15 mL) at a ratio of 4:1 (*v/v*) and subsequent shaking for 1 min using a vertical shaker. The top layer was collected and transferred into another centrifuge tube containing 4 g of calcined anhydrous sodium sulphate before centrifugation at 10,956 RCF and 4 °C for 10 min. The supernatant was decanted using glass wool while the liquid portion was placed in a glass tube. The mixture was further evaporated to dryness at 40 °C using a heating block (Barnstead Lab-line, Melrose Park, USA) under nitrogen gas flow. The remnant was then reconstituted with 450 µL of ethyl acetate and 50 µL triethylamine. The solution was finally transferred into an amber vial with an insert and stored at −20 °C before GC-MS analysis. Duplicate analyses were carried out for each model system.

#### 2.3.2. GC-MS Analysis

Calibration standards and brominated samples were analyzed using Agilent Technologies GC 6980 series fitted with a mass spectrometry detector 5973 Series and HP-Innowax capillary column (30 m × 0.25 mm, i.d., 0.25 mm film thickness (Agilent Technologies, Palo Alto, CA, USA). An aliquot of 2 µL was first injected in a split-less mode (split flow of 60 mL/min) at 250 °C using an auto-sampler (Agilent Technologies Series 7683, Palo Alto, CA, USA) with a purge activation time of 1 min. Helium was selected as a carrier gas set at a flow rate of 1.6 mL/min. The column was fixed at 65 °C for 1 min and gradually programmed at 15 °C/min to 170 °C, followed by 5 °C/min to 200 °C and 40 °C/min to 250 °C before held for 15 min at 250 °C. The selected ion monitoring (SIM) mode was used, and the m/z 149 for 2-bromopropenamide and 154 for 2-bromo(13C3)propenamide ions were monitored throughout the instrumentation process.

#### 2.3.3. Quantification of Acrylamide

Acrylamide content in all the model systems was quantified using ion at m/z 149 for 2-bromopropenamide and m/z 154 for 2-bromo(13C3)-propenamide. Different concentrations of acrylamide standards were used to construct the calibration curves. The equation obtained from the calibration curve was used to calculate the acrylamide level for the model systems. A linear calibration curve obtained was *R*^2^ > 0.996, while the limit of detection (LOD) was 8 µg/kg. The recovery was in the accepted range varying from 96.12% to 106.23%.

### 2.4. Statistical Analysis

Analysis of variance (ANOVA) was performed for all results while Tukey’s multiple comparison test assessed the means. The rate of acrylamide promotion is calculated using the following equation:

(1) $$Rate\;of\;acrylamide\;promotion\;\left(\%\right)=\frac{\left(\;acrylamide\;in\;homologue\right)-\left(acrylamide\;in\;control\right)}{acrylamide\;in\;control}\times 100\%$$

Linear regression was performed to predict the relative contribution of each type of homologue towards acrylamide formation. Parameters such as the slope (Beta) and the intercept of linear regression, besides the coefficient of regression (*R*^2^) and *p*-value, were estimated. Possible violations of the model assumptions were also examined. *p*-value, which is less than or equal to 0.05, was contemplated as statistically significant. Minitab Statistical Software 17 was used to perform all statistical analyses (Minitab Inc., State College, CA, USA).

## 3. Results and Discussion

Table 1 shows the profile of acrylamide concentration in asparagine-glucose model system at different levels of Vitamin A and E homologues. Acrylamide content in the control model system was measured at 2250 µg/kg. In comparison to the control experiment, fixing the homologue level at 1 µmol yielded a significant increase (*p* ≤ 0.05) in acrylamide for the model systems containing AT, DT, and GTT, respectively. The incorporation of BC, ATT and DTT also resulted in a considerable increase in acrylamide even though their differences were found insignificant. It is also noted that AT gave the highest acrylamide content, followed by DT and GTT. Except for DT, acrylamide concentration significantly increased (*p* ≤ 0.05) for all homologues at 10 µmol.

**Figure 1:**
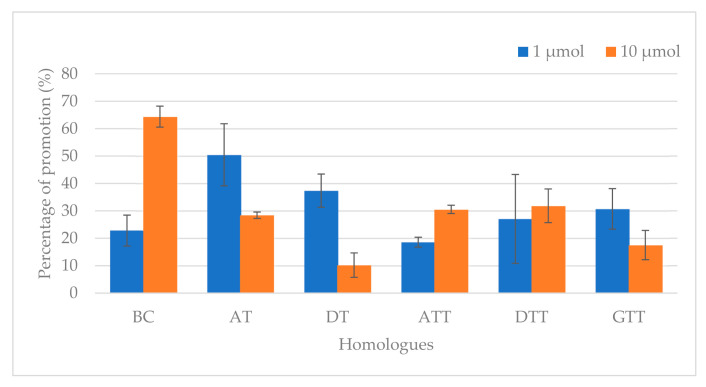
Rate of acrylamide promotion for different Vitamin A and E homologues at 1 and 10 µmol. Value are the means ± SD of duplicate experiments with duplicate determinations.

The rate of acrylamide promotion was calculated to predict the impact of individual homologue on the occurrence of acrylamide when subjected to heating (Figure 1). Regardless of homologue levels, a positive rate of acrylamide promotion was observed for all homologues. Moreover, the inclusion of BC into the model system displayed the highest acrylamide content while DT was the lowest. The model system fortified with AT showed the most remarkable rate of acrylamide promotion (50.5%) at 1 µmol; however, acrylamide promotion rate was paramount in BC (64.4%) upon endorsement of homologue level to 10 µmol. The effect of ATT (18.6%) and DT (10.2%) towards acrylamide promotion was the lowest when the homologue levels were set at 1 and 10 µmol, respectively.

Vitamin A and E homologues demonstrated some form of promoting effect on acrylamide formation in the model system compared to the unspiked system. The results are according to Tareke [15], who observed that Vitamin E promotes the accumulation of acrylamide in meat. The author also coined that Vitamin E can protect acrylamide from being eliminated during free radical initiated reactions. Some studies discussed the pro-oxidant effect of Vitamin E when increasing its concentration [16,22,23]. One prominent Vitamin E homologue, which is AT, can transport aqueous radicals due to alpha-tocopheroxyl radicals in its structure [22]. This could be the possible reason for the highest acrylamide content and rate of acrylamide promotion, particularly at 1 µmol. However, the study by Zeng et al. [17] showed the alternative observation where 0.5% of AT inhibits acrylamide formation in the fried snack model. The inconsistency in the effects of Vitamin E may be related to the complex function and chemical behavior of Vitamin E, being able to have an antioxidant, neutral, or pro-oxidant effect. Rietjens et al. [24] described the pathway to explain the dualistic behavior of AT that increased levels of AT (upon subsequent oxidative stress) result in increased levels of AT radicals. When other antioxidants do not efficiently scavenge AT radicals, they may act as reactive radical species themselves to initiate lipid peroxidation processes.

Table 1 also shows that acrylamide content in the model system coincided with the increase in BC. However, acrylamide content exhibited a contradictory trend when improving AT, DT, and GTT levels. The results demonstrated that each homologue gave different reactivity, which leads to their role either as an antioxidant or pro-oxidant. It has been reported that BC exhibited its pro-oxidant characteristics at high concentration and oxygen tension [25]. Conjugated double bonds present in the BC structure can be easily attacked by free radicals, which demonstrate its pro-oxidant behavior [26]. Indeed, BC enhances primary oxidative constituents [25], leading to acrylamide formation [27].

**Figure 2:**
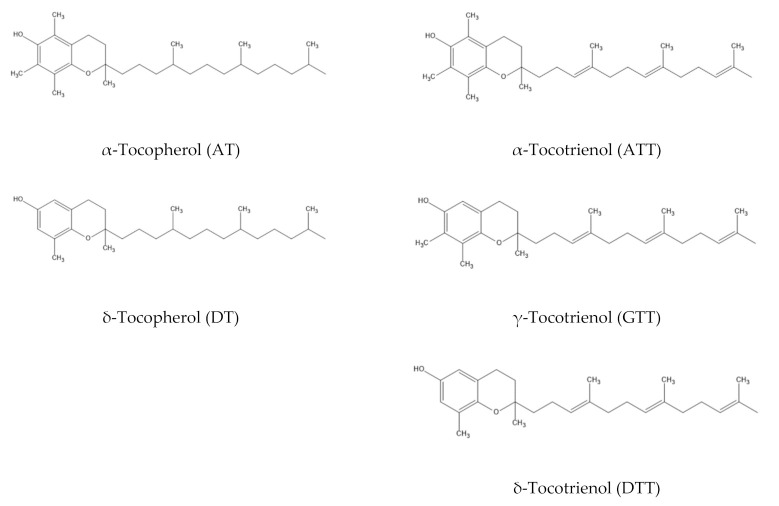
Chemical structures of different homologues of tocopherols and tocotrienols.

Virgin olive oil extracts promote acrylamide in potatoes, while oregano extracts demonstrated the alternative trend. The possible reason for this observation was that the disparity in the phenolic structures in both extracts resulted in different reactivity against acrylamide formation despite similar reaction conditions [28]. This basis supports our findings where the structural difference between tocopherols and tocotrienols (Figure 2) could contribute to different reactivity against acrylamide formation. Tocotrienols have the same methyl structures as those present in tocopherols except for three double bonds in the hydrophobic side chain [29]. Unlike most tocotrienols, enhancing the level of tocopherol led to a reduction of acrylamide (Table 1).

Other phytonutrients such as phenolic [30] and flavonoids [31] were also studied to investigate the promotion rate of acrylamide formation in a model system at different concentrations. Phenolic compounds are responsible for either shielding acrylamide from being destroyed or interacting with precursors during the Maillard reaction to inhibit the formation of acrylamide [32]. A moderate level of flavonoid could decelerate the formation of acrylamide. It can be conjectured that the amount of phytonutrients present in the model system determines their role as either anti- or pro-oxidant, which depends on the inherent properties of the phytonutrients and their reactivity against Maillard reactions [31].

It is clearly shown in Table 2 that contributions of AT (*R*^2^ = 0.652, *p* ≤ 0.05) and DT (*R*^2^ = 0.930, *p* ≤ 0.05) were significant to predict acrylamide formation. Both AT and DT gave negative contributions where the former was more prominent (Beta = −83.0) than the latter (Beta = −71.6). Dissimilarity in reactivity is the likelihood of structural difference between these homologues; AT consists of three methyl groups, whereas DT only has one methyl group present in its structure (Figure 2). Ubaldi et al. [5] emphasized that the presence of three methyl groups in AT resulted in higher reactivity as compared to DT. Thus, AT and DT, in their simplest form, could slow down the formation of acrylamide by improving the levels of both homologues.

## 4. Conclusions

This study demonstrated that Vitamin A and E homologues enhance acrylamide formation in the asparagine-glucose model system compared to control. Fixing the AT value at 1 µmol and BC at 10 µmol yielded the highest rate of acrylamide promotion. A significant increase in acrylamide content was observed with the increase in BC However, AT, DT, and GTT significantly reduced the acrylamide content with the increase in their level. Both AT and DT showed a significant pessimistic prediction against acrylamide formation, for which the latter gave a more prominent effect. In their simplest form, AT and DT played a crucial role in minimizing acrylamide formation when they increase in their level. Tocopherols and tocotrienols demonstrated different reactivity towards acrylamide content as a result of structural differences. Therefore, the amount of homologue added into the model system could determine their functionality either as an antioxidant or pro-oxidant. The influence of each homologue, in its simplest form, enables to induce acrylamide. For future studies, it is interesting to investigate the combined effect of mixed homologues on acrylamide formation.

## Figures and Tables

**Table 1:** Acrylamide concentration (µg/kg) of asparagine/glucose model system in control and with different concentrations of Vitamin A and E homologues. Value are the means ± SD of duplicate experiments with duplicate determinations.

	Acrylamide Concentration (µg/kg)	
Control ^1^	2250 ± 82 ^C^ [0 ppm]	2250 ± 82 ^E^ [0 ppm]
Homologue Levels	1 µmol	10 µmol
BC	2763 ± 95 ^BCb^ [537 ^2^ ppm]	3698 ± 73 ^Aa^ [5370 ppm]
AT	3385 ± 42 ^Aa^ [431 ppm]	2889 ± 45 ^BCb^ [4310 ppm]
DT	3091 ± 93 ^ABa^ [403 ppm]	2480 ± 81 ^DEb^ [4030 ppm]
ATT	2668 ± 37 ^BCa^ [425 ppm]	2937 ± 37 ^BCa^ [4250 ppm]
DTT	2859 ± 47 ^BCa^ [397 ppm]	2967 ± 21 ^Ba^ [3970 ppm]
GTT	2941 ± 69 ^ABa^ [411 ppm]	2644 ± 98 ^CDb^ [4110 ppm]

^1^ The model system that contained only a mixture of 10 µmol of each asparagine and glucose with the absence of any vitamin homologues served as control. Analysis was done in duplicate and the mean value was used in the analysis of variance to determine significant differences in acrylamide concentration among the control and treatments at different concentrations of Vitamin A and E homologues. ^2^ Values in the parentheses ([ ]) indicate the expression of homologue levels in ppm. Values within the same column with different uppercase letters (A–E) are significantly different (*p* ≤ 0.05). Values within the same row with different lowercase letters (a–b) are significantly different (*p* ≤ 0.05).

**Table 2:** Linear regression model for acrylamide formation at different levels of Vitamin A and E homologues.

Homologue	Beta	Intercept	^1^ *R* ^2^	*p*-Value
BC	91.7	2264	0.359	0.155
AT	−83.0	3468	0.652	*p* ≤ 0.05
DT	−71.6	3162	0.930	*p* ≤ 0.05
ATT	47.8	2460	0.584	0.133
DTT	40.6	2560	0.857	0.074
GTT	−33.0	2974	0.626	0.061

^1^*R*^2^ values are coefficients of regression.

## Data Availability

Data is contained within the article.
